# Prevalence and correlates of physical inactivity in adults across 28 European countries

**DOI:** 10.1093/eurpub/ckab067

**Published:** 2021-05-17

**Authors:** Katerina Nikitara, Satomi Odani, Nektarios Demenagas, George Rachiotis, Emmanouil Symvoulakis, Constantine Vardavas

**Affiliations:** 1 School of Medicine, University of Crete, Heraklion, Greece; 2 Department of Hygiene and Epidemiology, Medical Faculty, University of Thessaly, Larissa, Greece; 3 Department of Oral Health Policy and Epidemiology, Harvard School of Dental Medicine, Boston, MA, USA

## Abstract

**Background:**

Physical activity/inactivity is impacted by a plethora of intertwined factors. There are a limited number of studies on physical activity/inactivity that provide a European cross-country perspective. This study aims to present the prevalence and correlates of physical activity in adults across the 28 European Union (EU) member states.

**Methods:**

This is a secondary dataset analysis of the Special Eurobarometer 472 data on physical activity. The cross-sectional survey was conducted during December 2–11 in 2017 across 28 European countries. The data consisted of ∼1000 respondents aged ≧15 years per country. The current analysis was restricted to adults aged 18–64 years (*n* = 19 645).

**Results:**

More than one in three (36.2%, 95% CI: 35.1–37.3) adults in the EU were physically inactive, with substantial cross-country differences noted. Women were less likely than men to be adequately or highly physically active (aOR: 0.86, 95% CI: 0.78–0.95). Similarly, adults at the age of 40–54 (aOR: 0.65, 95% CI: 0.52–0.81) and 55–64 (aOR: 0.61, 95% CI: 0.49–0.77) were less likely to have moderate or high levels of physical activity in comparison with those 18–24 years of age. Finally, high SES was positively associated with physical activity (aOR: 1.4, 95% CI: 1.16–1.69).

**Conclusions:**

A notable percentage of adults in Europe are physically inactive. Further research is needed to elucidate the factors behind the cross-country differences and identify potential policy actions that may support adopting a physically active lifestyle and decrease the inequalities related to physical activity across Europe.

## Introduction

Physical activity is positively associated with health and quality of life.^1^ According to World Health Organization (WHO) recommendations (2020), healthy adults (18–64 years old) should undertake at least 150–300 min of aerobic physical activity in moderate-intensity or at least 75–150 min of vigorous-intensity aerobic physical activity every week.^2^ Notably, insufficient physical activity is one of the most important modifiable risk factors both for non-communicable diseases, including obesity, cardiovascular disease, diabetes, cancer, hypertension and osteoporosis/osteoarthritis.^3^ Physical inactivity is estimated to be responsible for ∼1 million deaths and 8.3 million disability-adjusted life-years annually in the WHO European Region.^3^ Furthermore, physical inactivity imposes economic costs of €80.4 billion per year to the EU-28, adding a significant financial burden to healthcare systems and societies.^3^ On the contrary, engaging in physical activity has a beneficial impact on the health of individuals, the economy and society.^4^ Despite the above benefits, previous research has noted that ∼23.4% (95% UI 20.9–28.0) of the central and eastern European population still does not meet the WHO recommendations for physical activity.^4^

Many personal, social and environmental factors have been associated with physical activity/inactivity. However, in Europe, the majority of such studies focus on specific subpopulations, such as children,^5^^,^^6^ adolescents,^7^^,^^8^ older adults^9^ and minority^10^ groups or focus on certain types of physical activity.^11–13^ Moreover, most studies that estimate the prevalence of physical activity/inactivity and its associated factors are either country-specific^14–17^ or international without a specific European focus, with studies reporting for European countries limited in number.^18^^,^^19^ With the above in mind, this study aims to present the prevalence and correlates of physical inactivity in adults across 28 European countries in 2017.

## Methods

### Data source

De-identified publicly available data were obtained from the Special Eurobarometer 472 on sport and physical activity. The cross-sectional survey was conducted in December 2017 across 28 European countries at the request of the European Commission, Directorate-General for Education Youth, Sport and Culture. The data collection included the UK as part of the European Union (EU), as it was performed before Brexit, however, we refer to European Countries rather than EU member states (EU MS) in the current document.

The Eurobarometer uses a multi-stage, random sampling design. For this, the number of sampling points is drawn with probability proportional to population size and population density, covering the whole territory of each country. In all countries, gender, age, region and size of the locality were introduced in the iteration procedure. A comparison was made between the sample composition and population distributions of each of the participating countries to adjust for non-response. Participants from different demographic groups were interviewed face-to-face at home in their first language. The data consisted of ∼1000 respondents aged ≧15 years per country, leading to a pooled sample size of 28 031. The analysis of this study was restricted to adults aged 18–64 years (*n* = 19 645).

### Measures

To maintain consistency with the previous secondary analysis on Eurobarometer data report by Gerovasili et al.,^19^ we assessed the frequency and duration of three types of physical activity based on the definition of physical activity derived from the International Physical Activity Questionnaire (IPAQ)^20^: vigorous activity, moderate activity (excluding walking) and walking. The IPAQ classification has been validated in several settings.^21^ The frequency was assessed with the questions ‘In the last 7 days, how many days did you do vigorous physical activity like lifting heavy things, digging, aerobics or fast cycling?’, ‘In the last 7 days, on how many days did you do moderate physical activity like carrying light loads, cycling at a normal pace or doubles tennis? Please do not include walking’, and ‘In the last 7 days, on how many days did you walk for at least 10 minutes at a time?’ Duration of physical activity was assessed with the following questions ‘In general, on days when you do [type of activity], how much time do you spend at it?’ Respondents reported duration in minutes or answered ‘never’ or ‘don’t know’. The response ‘never’ was treated as a numeric value of zero, and those who answered ‘don’t know’ to any of the relevant questions were excluded from the analysis (*n* = 261).

We also assessed metabolic equivalents of task (METs) to estimate total physical activity per week. Based on energy expenditure estimates used in the IPAQ, each type of activity was assigned a MET value as follows: 3.3 METs for walking, 4 METs for other moderate activity and 8.0 METs for vigorous activity. The total amount of MET-minutes (MET-min) per week was calculated for each respondent by multiplying the reported time spent doing each type of activity by corresponding MET.

Based on the IPAQ criteria, respondents’ physical activity level was classified into the following three categories: high (vigorous activity on at least 3 days achieving ≧1500 MET-min/week OR ≧7 days of any combination of walking, moderate activity achieving ≧3000 MET-min/week): moderate (≧3 days of vigorous activity and/or walking at least 20 min per day OR ≧5 days of moderate activity and/or walking at least 30 min per day OR ≧5 days of any combination of walking, moderate/vigorous activity achieving ≧600 MET-min/week: and low (not meeting high/moderate criteria).

### Statistical analysis

Data were weighted to be nationally representative of each of the participating countries. Descriptive statistics were computed for the 28 countries overall and by country with 95% confidence intervals (95% CIs). Multivariable logistic regression analysis was used to examine associations between physical activity and the following sociodemographic factors: country, sex, age, education, difficulty paying bills, urbanization, region and life satisfaction. Education was assessed as respondents’ age at which they stopped full-time education with the question ‘How old were you when you stopped full-time education?’ Difficulty paying bills was assessed with the question ‘During the last twelve months, how often have you had difficulties in paying bills at the end of the month…?’, and was regarded as a proxy of socioeconomical status (SES). Urbanization was self-reported to the question ‘Would you say you live in a…?’ We confirmed that variance inflation factors for all independent variables in the model were below 2.0. All analyses were conducted with R version 3.6.2.

## Results

### Physical activity level across the 28 EU MS

Concerning overall physical activity levels across the 28 European countries, 36.2% (95% CI: 35.1–37.3) of adult residents were classified as physically inactive, and the highest proportions were noted in Southern Europe. As presented in table 1, Portugal was found to have the highest prevalence of physical inactivity, with the percentage of individuals (18–64 years old) not meeting the IPAQ cut-offs for moderate/high physical activity reaching 63.7% (95% CI: 60.2–67.2) of the population. Slightly lower, but still elevated were the levels of physical inactivity in Malta, Italy and Cyprus. Conversely, Sweden had the lowest rate of physically inactive adults (19.2%, 95% CI: 15.3–23.1) followed by Germany, the Netherlands and Estonia, in which 21.1% (95% CI: 18.2–24.1), 22.5% (95% CI: 19–26) and 23.7% (95% CI: 19.9–2.5) adults, respectively, reported inadequate levels of physical activity. The total proportions for moderate and high levels of physical activity across the 28 European countries were estimated at 40.8% (95% CI: 39.7–42) and 23% (95% CI: 21.9–24), respectively. Significant geographical variability was also noted, with the highest proportions of moderate physical activity in Northern and Southern Europe, namely in Sweden (51.3%, 95% CI: 46.2–56.3), Spain (49.3%, 95% CI: 45.5–53.1) and Denmark (47.9%, 43.4–52.5) and the highest rates of high physical activity in Western and Northern Europe, such as Germany (37.7%, 95% CI: 34.1–41.4), Luxembourg (34.9%, 95% CI: 29.1–40.6) and Estonia (34.8%, 95% CI: 30.6–39.1).

**Table 1:** Level of physical activity of individuals aged 18–64 across the 28 European countries expressed in percentages, 2017 (*n* = 19 645)

Countries	*N*	Low	Moderate	High
		% (95% CI)	% (95% CI)	% (95% CI)
EU 28^a^	19 645	36.2 (35.1–37.3)	40.8 (39.7–42)	23 (21.9–24)
Southern Europe				
Croatia	844	42.2 (38.7–45.6)	38.6 (35.2–41.9)	19.3 (16.5–22)
Cyprus	328	55.2 (49.2–61.1)	27.8 (22.5–33.2)	17 (12.4–21.7)
Greece	729	44 (40.1–47.9)	40.2 (36.3–44.1)	15.8 (12.9–18.7)
Italy	830	55.6 (52.1–59.2)	36.4 (32.9–39.9)	8 (6–9.9)
Malta	300	62.8 (56.5–69.1)	27.2 (21.4–33)	10 (5.8–14.3)
Portugal	790	63.7 (60.2–67.2)	25.7 (22.5–28.8)	10.7 (8.3–13)
Slovenia	715	34.5 (30.9–38.2)	42.6 (38.8–46.4)	22.8 (19.5–26.2)
Spain	724	30.2 (26.8–33.7)	49.3 (45.5–53.1)	20.4 (17.4–23.5)
Western Europe				
Austria	819	38.8 (35.3–42.3)	37.5 (34–41)	23.7 (20.6–26.8)
Belgium	746	40.2 (36.4–44.1)	37.2 (33.4–41)	22.6 (19.2–25.9)
France	700	37.8 (34–41.5)	40.6 (36.7–44.4)	21.7 (18.4–25)
Germany	1056	21.1 (18.2–24.1)	41.1 (37.5–44.8)	37.7 (34.1–41.4)
Luxembourg	367	27 (21.8–32.2)	38.1 (32.5–43.8)	34.9 (29.1–40.6)
The Netherlands	647	22.5 (19–26)	46.8 (42.5–51.1)	30.7 (26.8–34.7)
Northern Europe				
Estonia	579	23.7 (19.9–27.5)	41.5 (37.1–45.8)	34.8 (30.6–39.1)
Denmark	581	25.2 (21.4–29)	47.9 (43.4–52.5)	26.8 (22.7–30.9)
Finland	574	26.8 (23–30.6)	45.7 (41.4–50.1)	27.5 (23.6–31.4)
Ireland	776	39.2 (35.6–42.9)	41.3 (37.6–45)	19.5 (16.6–22.4)
Latvia	727	24.6 (21.1–28.1)	41.1 (37.1–45.2)	34.2 (30.4–38.1)
Lithuania	615	35.6 (31.5–39.7)	40.3 (36–44.7)	24 (20.3–27.8)
Sweden	545	19.2 (15.3–23.1)	51.3 (46.2–56.3)	29.6 (24.9–34.2)
UK^a^	896	30.9 (27.4–34.3)	43 (39.2–46.7)	26.2 (22.8–29.5)
Eastern Europe				
Bulgaria	820	42.8 (39.2–46.3)	39.6 (36.1–43.1)	17.6 (14.8–20.4)
Czechia	806	35.9 (32.4–39.5)	43.8 (40.1–47.6)	20.2 (17.1–23.3)
Hungary	746	35.5 (31.8–39.2)	38.2 (34.3–42)	26.3 (22.8–29.9)
Poland	744	46.1 (42.1–50.1)	36.4 (32.4–40.3)	17.5 (14.2–20.8)
Romania	801	46.4 (42.8–50.1)	37.2 (33.7–40.7)	16.3 (13.6–19.1)
Slovakia	840	38.9 (35.3–42.5)	36.4 (32.9–40)	24.7 (21.4–28)

a This represents the 28 European countries before Brexit.

Across the 28 European countries, the mean total physical activity level per week was 1940 MET-min (95% CI: 1884–1996), from which 816 MET-min were attributed to vigorous physical activity, 488 MET- min (95% CI: 781–852) to moderate physical activity and 651 MET-min (95% CI: 634–668) to walking. Broad differences were noted across the included countries, with the mean weekly total physical activity levels ranging from 931 MET-min (95% CI: 842–1021) in Italy to 2861 MET-min (95% CI: 2656–3066) in Germany. Consistently with the previous findings, Northern and Western European countries, such as Latvia (1285 MET-min, 95% CI: 1125–1445), Estonia (1239 MET-min, 95% CI: 1077–1402) and Germany (1219 MET-min, 95% CI: 1088–1351) were found to be first in terms of vigorous physical activity level, while Southern European countries, namely in Malta (351 MET-min, 95% CI: 202–501), Italy (388 MET-min, 95% CI: 326–450) and Portugal (393 MET-min, 95% CI: 312–473) were holding the last places. Respondents from Germany with 844 MET-min (95% CI: 770–917), followed by Luxembourg with 776 MET-min (95% CI: 645–906) and the Netherlands with 735 MET-min (95% CI: 660–810) reported the most time in performing moderate physical activity, while the least was noted in Malta (173 MET-min, 95% CI: 113–233), Italy (188 MET-min, 95% CI: 161–215) and Portugal (259 MET-min, 95% CI: 215–303). Finally, adults in Estonia had the highest levels of walking (921 MET-min, 95% CI: 844–998) and those from Cyprus the lowest (351 MET-min, 95% CI: 289–413) (table 2).

**Table 2:** Mean MET-min per week of total, vigorous and moderate physical activity, as well as walking among individuals aged 18–64 in 28 European countries, 2017 (*n*=19 645)

Countries	Total physical activity mean MET-min	Vigorous activity mean MET-min	Moderate activity mean MET-min	Walking mean MET-min
	% (95% CI)	% (95% CI)	% (95% CI)	% (95% CI)
EU 28	1940 (1884–1996)	816 (781–852)	488 (469–507)	651 (634–668)
Southern Europe				
Croatia	1825 (1663–1987)	783 (679–886)	493 (437–548)	561 (516–606)
Cyprus	1364 (1112–1615)	653 (478–828)	362 (275–448)	351 (289–413)
Greece	1439 (1308–1571)	576 (490–663)	418 (369–468)	446 (408–485)
Italy	931 (842–1021)	388 (326–450)	188 (161–215)	366 (336–396)
Malta	935 (708–1162)	351 (202–501)	173 (113–233)	412 (334–491)
Portugal	1022 (894–1151)	393 (312–473)	259 (215–303)	387 (350–423)
Slovenia	2025 (1826–2225)	827 (704–951)	521 (454–588)	692 (633–750)
Spain	1933 (1767–2100)	711 (605–817)	380 (327–434)	844 (789–898)
Western Europe				
Austria	1766 (1618–1915)	799 (710–887)	468 (418–517)	512 (469–554)
Belgium	1902 (1727–2078)	782 (668–895)	607 (541–672)	514 (466–562)
France	1886 (1699–2072)	821 (695–947)	430 (370–490)	637 (583–692)
Germany	2861 (2656–3066)	1219 (1088–1351)	844 (770–917)	818 (760–876)
Luxembourg	2675 (2308–3043)	1081 (852–1310)	776 (645–906)	845 (743–948)
The Netherlands	2342 (2170–2515)	1032 (914–1151)	735 (660–810)	582 (528–637)
Northern Europe				
Estonia	2800 (2552–3047)	1239 (1077–1402)	672 (587–758)	921 (844–998)
Denmark	2080 (1914–2245)	719 (611–826)	669 (592–746)	707 (638–777)
Finland	2081 (1906–2256)	1011 (900–1121)	454 (398–510)	636 (584–688)
Ireland	1626 (1489–1762)	656 (575–737)	403 (354–453)	571 (528–615)
Latvia	2848 (2623–3073)	1285 (1125–1445)	709 (626–792)	860 (797–923)
Lithuania	2139 (1929–2348)	920 (780–1059)	531 (458–605)	699 (630–767)
Sweden	2324 (2121–2528)	1125 (978–1273)	568 (492–645)	634 (571–698)
UK	2192 (2012–2372)	881 (768–993)	500 (439–561)	826 (764–887)
Eastern Europe				
Bulgaria	1524 (1381–1668)	598 (506–691)	365 (313–417)	591 (548–635)
Czechia	1883 (1714–2053)	806 (697–916)	458 (401–516)	633 (581–686)
Hungary	2173 (1976–2370)	1066 (929–1204)	602 (537–668)	511 (463–560)
Poland	1542 (1365–1718)	683 (573–793)	385 (329–441)	512 (461–564)
Romania	1614 (1440–1788)	647 (541–754)	340 (288–392)	645 (592–698)
Slovakia	1935 (1749–2120)	914 (800–1028)	441 (388–494)	633 (581–684)

### Physical activity level and sociodemographic factors

An adjusted logistic regression analysis identified that adequate (moderate and high) levels of physical activity were independently associated with sociodemographic factors (table 3). Women were less likely than men to be adequately or highly physically active (aOR: 0.86, 95% CI: 0.78–0.95). Similarly, adults between the age of 40–54 (aOR: 0.65, 95% CI: 0.52–0.81) and 55–64 (aOR: 0.61, 95% CI: 0.49–0.77) were less likely to have moderate or high levels of physical activity in comparison with respondents aged between 18–24 year of age. Moreover, residents in Eastern and Southern European countries had higher odds of being physically inactive compared to Northern European countries (aOR: 0.64, 95% CI: 0.55–0.74 and aOR: 0.61, 95% CI: 0.52–0.71), while, high SES was positively associated with physical activity (aOR: 1.4, 95% CI: 1.16–1.69).

**Table 3:** Association between sociodemographic factors and being adequately or highly active among individuals aged 18–64 in 28 European countries, 2017 (*n*=19 645)

		*N*	% (95% CI)	AOR
Overall		19 645	63.8 (62.7–64.9)	–
Gender	Man	8838	65.8 (64.2–67.4)	ref.
	Woman	10 807	61.8 (60.3–63.3)	0.86 (0.78–0.95)^*^
Age	18–24	1862	73.2 (70.2–76.3)	ref.
	25–39	5792	67.4 (65.5–69.2)	0.85 (0.68–1.05)
	40–54	6935	60.1 (58.2–62.1)	0.65 (0.52–0.81)^*^
	55–64	5056	57.9 (55.6–60.1)	0.61 (0.49–0.77)^*^
Education	15 years	1731	53.2 (49.6–56.8)	ref.
	16–19 years	9305	60.7 (59–62.3)	1.09 (0.92–1.29)
	20+ years	7156	69.1 (67.3–70.8)^*^	1.36 (1.13–1.62)^*^
	Still studying	1129	75.4 (71.5–79.2)	1.53 (1.12–2.09)^*^
Difficulties paying bills	Most of the time	1892	52.2 (48.5–56)	ref.
	From time to time	5559	58.2 (56.1–60.3)	1.2 (0.99–1.45)
	Almost never/never	11 848	67.8 (66.4–69.1)	1.4 (1.16–1.69)^*^
Perceived urbanization	Rural village	5502	64.8 (62.7–66.8)	ref.
	Small/mid-size town	8436	62.6 (60.9–64.3)	0.9 (0.79–1.01)
	Large town	5696	64.7 (62.7–66.7)	0.97 (0.85–1.11)
EU region	Northern	5293	70.5 (68.1–72.9)	ref.
	Western	4335	70.9 (69–72.9)	1.11 (0.95–1.29)
	Eastern	4757	56.9 (54.9–58.9)	0.64 (0.55–0.74)^*^
	Southern	5260	54.3 (52.2–56.3)	0.61 (0.52–0.71)^*^
Life satisfaction	Very satisfied	4411	74.1 (72–76.2)	ref.
	Fairly satisfied	11 915	62.8 (61.5–64.2)	0.74 (0.64–0.84)^*^
	Not very satisfied	2677	52.9 (49.9–56)	0.6 (0.5–0.73)^*^
	Not at all satisfied	486	49.6 (42.6–56.5)	0.53 (0.38–0.73)^*^

AOR, adjusted odds ratios controlling for all factors in the table.

* Statistically significant results (*P*<0.005).

## Discussion

Our study, based on the updated data of Eurobarometer 2017, indicated that approximately one-third (36.2%) of the adult population under the age of 65 was physically inactive across 28 countries in Europe. In 2013, Gerovalili et al.^19^ within a secondary data set analysis of the previous Eurobarometer noted a lower prevalence of physically inactive adults (28.6%), suggesting a potential increase in physical inactivity between 2013 and 2017 as indicated by our analyses. Likewise, we found a lower total MET-min per week in 2017, of 1940 MET-min, compared to the 2151 MET-min in 2013.^19^ However, it is important to note that different cut-off criteria with regard to physical activity levels were used between the two studies, and hence this should be taken into account when comparing data. Similarly, Mayo et al.^22^ also noted that the prevalence of physical inactivity had increased between 2013 and 2017 in Europe, even though different cu-offs of physical activity were used and these results further agree with a pooled analysis of population-based surveys in which the prevalence of physical inactivity in Central and Eastern Europe and high-income Western countries gradually increased during this timeframe.^23^

With regard to physical activity levels, a substantial cross-country differences were noted, which were expected due to the variability of geographical, sociodemographic and cultural characteristics between the 28 European countries. Southern and Eastern European countries found to be more physically inactive in comparison with Northern and Western European ones. This could be partly explained by the positive association between country prevalence of sedentary behaviour and country gross domestic product per capita in adults, which has been elucidated within a previous European study,^24^ or potentially to other climate or lifestyle factors. The quality of the environment and the built environment itself are also significant factors that seem to affect the levels of physical activity in the European adult population.^25^ Finally, variations in policy orientations and investments concerning public health across countries may affect the weight that different countries give to promotion of physical activity and, subsequently, the population’s physical activity behaviour.^26^ However, it is important to note that in a recent WHO International Inventory of national policies and documents for promotion of physical activity, a national policy document on physical activity promotion was reported in 28 European Countries.^27^ The impact of income on physical activity in an individual level, was also presented in our study. Interestingly, the respondents of low SES had a higher likelihood of being physically inactive, as shown in similar previous studies.^27^ Nevertheless, it should be noted that physical activity includes four pillars: occupational, domestic, transportation and leisure time. A recent systematic review indicated that the association between physical activity and SES is mostly a relationship between Leisure-Time Physical Activity and high SES.^28^ Although we assessed the total level of daily physical activity, without a distinction between occupational and leisure-time physical activity, Our results may support this hypothesis as we did identify an association between high SES and elevated physical activity. Finding strategies to counteract the imbalance in physical activity levels across SES groups remains among the biggest challenges within physical activity policy.

Another interesting finding of this study and consistent with the current literature is that gender and age are significantly associated with physical activity.^18^ Females and older adults had a higher likelihood of being physically inactive in comparison to males and younger adults, respectively. While a parallel study assessing the gender gap in physical inactivity in Europe using the 2013 and 2017 Eurobarometer datasets also noted a higher prevalence of PIA was observed in women for 2017, and for 2013.^22^ However, a cross-national European study focussed on sex differences of leisure-time physical activity, concluded that in countries with a higher level of equal opportunities between women and men in many life aspects, sex was not correlated with leisure-time physical activity.^29^^,^^30^ These results may show that sex-based inequality could be a possible explanation from a societal perspective. Moreover, in many countries, the women’s social role includes responsibilities including childcare and household managing, limiting the possibility of leisure-time physical activity, while it is more likely for men to participate in associations and groups where physical activity is promoted.^31^ With regard to age and physical activity, numerous studies have been conducted in order to detect the barriers and motivators for physical activity in older adults, with the most dominant ones referring to perceived physical and mental health.^32^

Finally, a strong correlation was found in this study between life satisfaction and physical activity, where adults with a high self-reported life satisfaction were more likely to be adequately or highly active. Although the majority of scientific evidence shows the positive impact of physical activity on life satisfaction, a reversed relationship between those two variables is also possible. A study by Schnohr et al.^33^ found increased life satisfaction in joggers with increased physical activity intensity. Additionally, Valois et al.^34^ found inadequate physical activity levels to be associated with low life satisfaction.

The strengths of this study include the representativeness of the sample in each of the 28 European countries as occurs from the Eurobarometer sampling methodology^1^ and the use of the same questionnaire, which allowed us to make cross-country comparisons. On the contrary, our study has several limitations, which should be taken into account prior to the interpretation of the results. First, this study is questionnaire based and there is a potential for information bias to occur (e.g. recall and social desirability bias). Second, we employed a cross-sectional, descriptive design and it is impossible to establish causality between cause and effect. Moreover, potential limitations arise from the fact that only approximate data on each individual’s activity levels could be collected due to the questions that were included within the Eurobarometer survey.

## Conclusions

Conclusively, our results noted that approximately one in three adults under 65 across the 28 European countries was physically inactive. Furthermore, we identified several individual/demographic (sex, age group, education) and macro-environment (economic difficulties, geographic setting) risk factors for physical inactivity. In particular, in terms of geographical factors, we report a considerable North/South East Europe divide in physical exercise outcomes. Moreover, as region/country specific differences were noted across the 28 European Countries, further research is needed to elucidate the factors behind these differences and to identify potential policy actions that may support the adoption of a physically active lifestyle across Europe.

*Conflicts of interest*: None declared.

**Key points**

- More than one in three of adults in 28 European countries were classified as physically inactive.
- Women were less likely than men to be adequately or highly physically active.
- Adults at the age of 40–54 and 55–64 were less likely to have moderate or high physical activity.
- High SES was positively associated with physical activity.
